# Pilocarpine in the Treatment of Ascites Caused by Disease of the Liver

**Published:** 1884-10-20

**Authors:** 


### PILOCARPINE IN THE TREATMENT OF ASCITES CAUSED BY DISEASE OF THE LIVER.

Ascites, caused by cirrhosis of the liver, has not been very amenable to treatment up to this time. Most clinicians have, therefore, come to the conclusion that paracentesis was the only resort. Drastics and diuretics had to be abandoned as inefficient, and, in fact, there seemed to be little hope left for this class of sufferers. Harley and Roberts claim that repeated paracentesis promises good results, while, on the other hand, there are those who have never accomplished anything by puncture. Mackenzie reports two cases as cured by means of flannel bandages tightly wrapped around the abdomen. Siegnit combines with the bandaging faradization of the abdominal muscles. From all the different reported cures one can readily understand that the therapy of ascites, caused by disease of the liver, has been quite unsatisfactory, and most of the cases have been treated on the expectant plan.

During the year of 1880 the author treated a case of this kind by all the remedies that have been recommended in the books without any results. Finally paracentesis was resorted to, but with each operation matters got worse and the quantity of liquid steadily increased. It occurred to him to administer pilocarpine immediately after an operation, to prevent or give other course to the serous exudation that, each time, was emptied into the peritoneal cavity. The patient received twice daily 0.015 (gr. $\frac{1}{4}$) of pilocarpine for six consecutive days with the effect of causing profuse diaphoresis and discharge from the salivary glands. Stimulants (whisky, 60 grammes [℥ ij]) were given the patient to counteract the weakening action of this medication. From the administration of the first dose the ascites diminished, and the patient was finally discharged from the hospital as cured. Three months later the patient was examined, but did not discover a symptom indicating disease of the liver or ascites.

Another case the author treated in conjunction with Dr. Haas. The diagnosis was an enormous ascites caused by cirrhosis of the liver. The swelling was so great that it caused dyspnœa, for the relief of which paracentesis was made a few times and 20 to 30 liters (5 to 8 gals.) fluid removed. The effect was not lasting. Finally respirations ran 38 and the pulse 118 per minute. The urinary secretion was entirely suppressed. After a consultation it was concluded to employ large doses of pilocarpine, since the case seemed hopeless. The patient received twice daily 0.01 gramme (gr. 1/6) of pilocarpine in a large dose of whisky, besides some solid food. The drug did not seem to act so favorably in this case, diaphoresis being very scanty. Two weeks later, after another puncture, the dose of pilocarpine was increased to 0.02 (gr. 1/3) and followed by a large quantity of whisky, with the result of causing profuse sweating. Pulv. jalap, with tart. depuratus, was given in conjunction for the obstipation. The patient gradually recovered, when he was discharged, and he was as healthy as before.

If the good results obtained in these two cases do not indicate much, they may cause other observers to try the pilocarpine treatment. Paracentesis often does not only do no good, but seems to do harm. Both of these patients were very much weakened. and it seems almost incredible that they could tolerate 0.02 grammes (gr. 1/3) of pilocarpine twice a day, but possibly the large doses of stimulants given shortly after did much to obviate the depressing effects of the pilocarpine drug.—*Mittheilungen des Vereins der Aerzte.*—*Ther. Gaz.*

